# Pathogen population bottlenecks and adaptive landscapes: overcoming the barriers to disease emergence

**DOI:** 10.1098/rspb.2016.0727

**Published:** 2016-08-31

**Authors:** Jemma L. Geoghegan, Alistair M. Senior, Edward C. Holmes

**Affiliations:** 1 Marie Bashir Institute for Infectious Diseases and Biosecurity, Charles Perkins Centre, School of Life and Environmental Sciences and Sydney Medical School, The University of Sydney, Sydney, New South Wales 2006, Australia; 2 School of Mathematics and Statistics and Charles Perkins Centre, The University of Sydney, Sydney, New South Wales 2006, Australia

**Keywords:** virus, evolution, adaptation, emerging diseases, spillover, influenza

## Abstract

Emerging diseases are a major challenge to public health. Revealing the evolutionary processes that allow novel pathogens to adapt to new hosts, also the potential barriers to host adaptation, is central to understanding the drivers of disease emergence. In particular, it is unclear how the genetics and ecology of pathogens interact to shape the likelihood of successful cross-species transmission. To better understand the determinants of host adaptation and emergence, we modelled key aspects of pathogen evolutionary dynamics at both intra- and inter-host scales, using parameter values similar to those observed in influenza virus. We considered the possibility of acquiring the necessary host adaptive mutations both before (‘off-the-shelf’ emergence) and after (‘tailor-made’ emergence) a virus is transmitted from a donor to a new recipient species. Under both scenarios, population bottlenecks at inter-host transmission act as a major barrier to host adaptation, greatly limiting the number of adaptive mutations that are able to cross the species barrier. In addition, virus emergence is hindered if the fitness valley between the donor and recipient hosts is either too steep or too shallow. Overall, our results reveal where in evolutionary parameter space a virus could adapt to and become transmissible in a new species.

## 1. Introduction

Emerging pathogens that cross the species barrier to infect new hosts pose an important threat to human and animal health. Despite the high morbidity and mortality due to some emerging infections, the evolution of sustained transmission cycles in a novel host following a species jump is a relatively rare occurrence. Rather, many, if not most, emergence events in reality reflect isolated ‘spillover’ events in which a pathogen causes infection in a new host but without large-scale onward transmission. An informative example is provided by the highly pathogenic H5N1 subtype of avian influenza A virus. Although this virus is primarily associated with outbreaks in wild and domestic birds (i.e. poultry), since 2003 it has caused over 800 laboratory-confirmed cases in humans in 16 countries, with more than 400 deaths [1]. Despite these repeated ‘spillover’ events, H5N1 has not been able to evolve sustained human-to-human transmission, likely because of a lack of airborne transmission. Experimental studies suggest that H5N1 may only require five amino acid substitutions across the viral genome to become transmissible, between ferrets, by respiratory droplet or aerosols [2]. Although it is uncertain whether these same mutations will lead to productive infections in humans, or if other evolutionary pathways can be followed, the emergence of a novel airborne virus such as H5N1 to which humans are immunologically naive and highly susceptible would clearly be of major public health importance [3].

**Figure 1.**
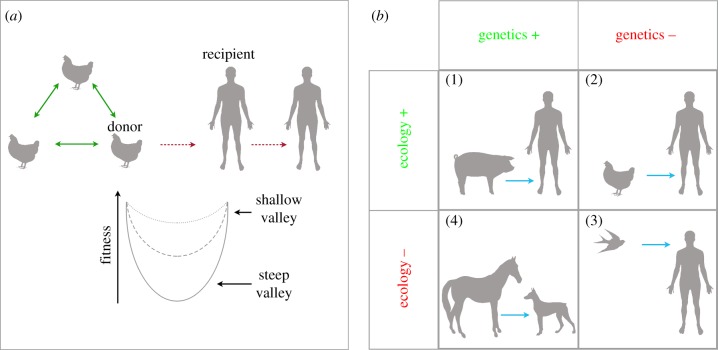
(*a*) Virus transmission among donor hosts, and between a donor and recipient host of different species. The adaptive landscape shows that viral fitness is maximized within each host species but that a fitness valley must be overcome during inter-host transmission. (*b*) The dual roles of genetics and ecology in virus emergence. Each box exemplifies the transmission of a virus from a donor to a recipient species. Panel 1: swine and human; panel 2: poultry and humans; panel 3: wild birds and humans; and panel 4: equine and dogs. (Online version in colour.)

To establish a productive infection in a new host a virus must overcome multiple evolutionary and ecological barriers [4]. From an evolutionary perspective, these barriers can be described in terms of an ‘adaptive landscape’ in which species jumps are depicted as a fitness valley. Critically, the more phylogenetically divergent the host species, then on average, the steeper the fitness valley [5]. Fitness valleys are likely to be especially steep in the case of host range because mutations that optimize a virus' ability to infect a (new) recipient host will, at the same time, often be deleterious for the virus in the (original) donor host (figure 1*a*) [6,7]. As a case in point, Herfst and colleagues used a combination of site-directed mutagenesis and serial passage to enable the H5N1 virus to acquire the five mutations necessary for airborne transmission in ferrets [2]. Although surveillance data have revealed that two of these mutations are already present in avian H5N1 viruses [3,8,9], their collective absence (particularly within the same animal) implies that they are deleterious, especially in combination. Hence, the successful adaptation of H5N1 to mammalian transmission cycles represents a steep fitness valley that has yet to be crossed. In addition, even if a virus is able to acquire the necessary genetic modifications it must also adapt to accommodate the behaviour and ecology of the recipient species to enable its sustained transmission. This fundamental interplay between genetics and ecology can be illustrated by the following examples observed in nature (figure 1*b*):

*Panel 1*. The genetic and ecological barriers to successful emergence are seemingly low in the case of influenza A virus in pigs and humans. The relationship between swine and human influenza was noted during the global influenza pandemic of 1918–1919 [10], and serological surveillance has revealed the presence of human influenza viruses in swine populations worldwide [11]. Importantly, swine are susceptible to both avian and human influenza viruses and as such have been hypothesized to act as ‘mixing vessels’ for novel virus strains [12], although more recent work suggests that pigs may be more at risk of human infections than the reverse [13] and hence may not be evolutionary ‘intermediates’ [14]. Successful ‘ecological adaptation’ of a swine virus to a recipient (human) host occurred during the 2009 H1N1 swine influenza pandemic, which resulted in an estimated 100 000–400 000 human deaths worldwide [15].
*Panels 2 and 3*. While birds are likely the natural reservoir for influenza A viruses, transmission from birds to mammalian species, including humans, is relatively rare. Although occupational exposure to poultry seemingly provides ample ecological opportunity for adaptation, avian influenza virus generally results in dead-end spillover events in which onward transmission among humans is limited (or non-existent) because the virus lacks the necessary mammal-specific mutations. The recent appearance of highly pathogenic H5N1 and H7N9 influenza viruses, characterized by multiple spillovers into humans but little or no onward transmission, present informative cases of this effect [16].
*Panel 4*. Equine-derived H3N8 canine influenza virus (CIV) is an example of a virus that appears to be genetically well adapted to transmission among dogs, but ecologically constrained due to the lack of sufficient host contact networks. This has resulted in a highly patchy distribution, characterized by sporadic and short-lived outbreaks in the United States, often confined to animal shelters [17], but a marked lack of sustained transmission in most (domestic) dog populations that may be too small and sparse to support ongoing transmission. Hence, although a pathogen may possess all the mutations required to successfully infect a new host, whether it establishes itself is also dependent on the underlying population ecology. Interestingly, a new H3N2 CIV of avian origin has emerged in Asia and, more recently, in North America [18]. Whether, or how, this virus differs in transmissibility from the H3N8 variant is currently unknown. As dogs are susceptible to both mammalian (equine) and avian influenza viruses, they, like pigs, have the potential to become mixing vessels, although there is no evidence that this has happened to date [14].

An additional factor that may act as a barrier to viral emergence is that most forms of transmission involve a major population bottleneck, in which a limited number of randomly sampled individuals (virions) create a new founding (inoculum) population. Such a sharp reduction in population size can severely reduce genetic variation and hence is likely to have profound effects on the population structure of the evolving virus [19]. Unfortunately, formal estimates of bottleneck size for virus transmission between animal hosts in nature are limited. For example, many studies suggest that the founding virus population for HIV is a single genotype for both vertical and horizontal transmission [20], and between one and two genotypes have been estimated to initiate new HCV infections [21]. These estimates rely on measuring changes in the extent of genetic diversity between the infected source and the new infection. Crucially, however, the actual number of *transmitted virions* in natural infections, as opposed to the number of transmitted genotypes, remains largely unknown. Somewhat more is known about bottleneck size during virus transmission among plants, where experimental manipulation is easier. In these cases, estimates also predict an extremely narrow bottleneck of between 0.5–3.2 virions for insect-borne transmission [22] and 1.3–3.3 virus particles for leaf-contact transmission [23].

Transmissibility is a key component of host adaptation, and hence virus fitness, that should be optimized by natural selection. However, whether the mutational changes that underpin this trait are fixed before or after a virus enters a new host species is unclear, particularly given the nature of fitness landscapes described above. Here, we explore the likelihood of successful adaptation of a virus that jumps from a donor to a recipient species, incorporating a variety of fitness landscapes and population bottlenecks of differing magnitude. In particular, we consider two specific models of viral emergence. In the first, termed the ‘off-the-shelf’ model [24,25], host adaptation occurs when the virus population transmitted from the donor to the recipient contains, by chance, all of those mutations required to successfully adapt to a new host species without the need for further mutation [26]. As an alternative, we investigate the likelihood of emergence under a ‘tailor-made’ model [24,25], in which viruses may adapt (i.e. generate and potentially fix beneficial mutations) in the recipient, thereby enabling successful onward transmission in the new host species. By modelling intra- and interhost virus evolutionary dynamics, and exploring a diverse range of parameters for fitness and transmission events, we aim to come to a better understanding of how such factors may impact the emergence of novel viruses.

## 2. Material and methods

### (a) Model overview

To determine the likelihood of a virus accumulating the necessary mutations to successfully cross a species barrier, and hence the probability of disease emergence in a novel host, we used an agent-based model (ABM) with a genetic algorithm. Our ABM simulates the intra- and interhost evolutionary dynamics of a virus population through time using parameters broadly analogous to those from H5N1 influenza virus. Our model assumes that a donor host is infected with a virus (agents) and experiences approximately 5 days of infection before the virus is transmitted to a recipient host of a different species. This recipient host itself then experiences an infection also lasting approximately 5 days, before we quantify the status of the infection (i.e. the number of virions and their level of adaptation).

### (b) Virus genome and fitness

**Figure 2.**
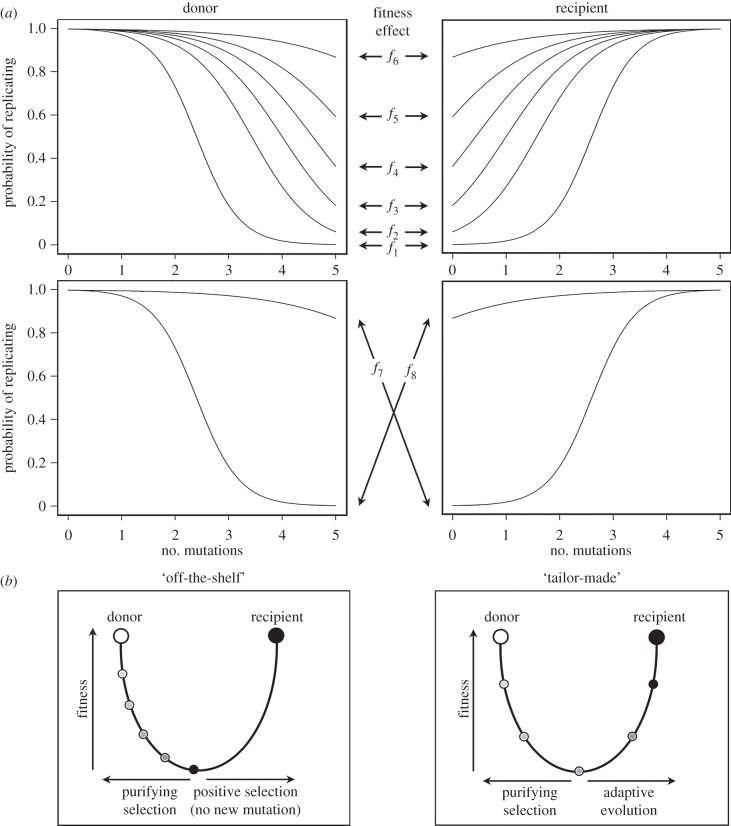
(*a*) Fitness is defined by the probability of reproducing as a function of the number of mutations (ranging from 0 to 5). Eight different scenarios are investigated (*f*_1_–*f*_8_), reflecting (i) the fitness valley becoming progressively shallower between two host species: the donor and the recipient (top panels), and (ii) different fitness effects between the two hosts (bottom panels) such that the virus is a ‘generalist’ in either the donor (*f*_7_) or the recipient (*f*_8_). (*b*) The adaptive landscapes of host adaptation and emergence. Under the ‘off-the-shelf’ model, mutation only occurs in the donor host and genetic variants are transmitted to the recipient host by chance. In the ‘tailor-made’ model, the virus may accumulate further adaptive mutations in the new host during the course of infection. This figure was inspired by Holmes [5] and Kuiken *et al.* [6].

We assume a haploid virus genome with host adaptation determined by five sites (i.e. fitting the parameter values identified by Herfst *et al*. [2]). Therefore, each virion has a genotype with five loci, each of which may be either wild-type (0) or mutated (1). The probability of a locus mutating from one state to the other (*m*; all parameters and variables are given in electronic supplementary material, table S1) is equal across virions, loci and states. We use a mutation rate that is typical for influenza viruses, at 10^−5^ mutations per site, per genome replication [27]. Analogous to a two-allele five-locus haploid model, a total of 2^5^ = 32 possible genotypes exist. However, for simplicity, we assume that virions with *n* mutations have equivalent fitness regardless of their position in the genome. A virion's fitness is defined by its probability of replicating and producing progeny, which is a function of the number of mutations carried and the host in which a virus finds itself. We explored eight different functions of genotype (number of mutations) on fitness (probability of replicating; figure 2*a*). In terms of the fitness landscape described earlier, this can be envisaged as a measure of the slope of the fitness valley, in which a severe fitness effect reflects a steeper fitness valley between hosts. The fitness functions, therefore, ranged from very steep to progressively more shallow, which are referred to as *f*_1_ to *f*_6_. We also considered two additional fitness functions, *f*_7_ and *f*_8_, that reflect differences in fitness effects between the hosts (figure 2*a*; electronic supplementary material, table S1). Under *f*_7_, viral mutations in the donor host have very little effect on fitness (equivalent to *f*_6_), but a large effect once transmitted to the recipient host (equivalent to *f*_1_). By contrast, *f*_8_ represents the opposing case (i.e. *f*_1_ in the donor and *f*_6_ in the recipient).

### (c) The donor

The model is initialized with a (founding) population of 10 virions, comprising only the wild-type genotype. The model is then run for 20 iterations in this host, analogous to a typical round of influenza replication lasting 6 h over a 5-day infection. Although there is a broad consensus that duration of replication for influenza is approximately 6–8 h [28,29], we recognize that this might vary such that peak viral titres in some cell types are achieved at approximately 12 h. For this reason, we later varied this parameter, running the model for both 15 and 10 viral generations, which is analogous to the duration of replication lasting 8 and 12 h, respectively. On each iteration of the model, each virion (i) survives and (ii) replicates with a given probability. Accordingly, one iteration is equivalent to one viral generation. The probability that a virion survives generation-to-generation is density-dependent and is implemented as a logistic regression where survival is predicted by density as given by:2.1
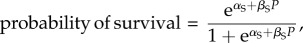
where e is the natural exponent; *α*_S_ is the log odds probability of surviving when the density is 0 (i.e. the intercept in the regression); *β*_S_ is the effect of density on the log odds of survival (i.e. the slope) and *P* is the population density (total number of virions divided by the carrying capacity, which is fixed at 10^8^ in all simulations). In all simulations we assume a negative slope, such that the probability of survival decreases as *P* increases. The survival probability of all virions is equally affected by density, and all parameters relating to density dependence were fixed throughout model runs. We selected parameters that allow steady population growth and then keep the population size at approximately 10^8^ after the replication step once the carrying capacity is reached.

Replication involves a ‘parental virion’ giving rise to *r* new virions, with *r* set to 16 here (from a practical programming perspective the original ‘replicating’ virion is then removed from the simulation). Each virion arising from a replication inherits the ‘parent's' genotype, with mutation. The probability of a virion replicating on an iteration in the donor is a function of the number of mutations carried. Again, we implement this as a logistic regression, as given by:2.2
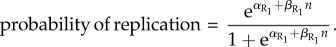
where e is as above; 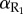 is the log odds probability of reproducing when a virion carries no mutations in the donor; 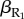 is the effect of mutation on the log odds of reproducing in the donor and *n* is the number of mutations the virion carries. We explored negative 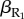 values such that as *n* increases the virion has a reduced probability of replicating, and we varied the severity of this effect (exact values were selected to yield the fitness functions in figure 2*a*; see electronic supplementary material, table S1), equivalent to assuming that virus genomes with no mutations are better suited to the donor host.

### (d) Transmission between hosts and the population bottleneck

After 20 generations in the donor (equivalent to approximately 5 days of infection) the host changes, analogous to a shift in host species. We assume that the population experiences a bottleneck during this transmission event. Hence, from the existing virus population *b* randomly selected virions were chosen as founders for the new recipient host and the remaining population is disregarded. Because formal estimations of bottleneck size for virus transmission between animal hosts in nature are limited, we explored a wide range of values for *b* (electronic supplementary material, table S1).

### (e) The recipient

As above, the virus infects a new host species following a population bottleneck. Again, the model iterates over 20 viral generations. The probability of a virion surviving generation-to-generation is identical in the two hosts (equation (2.1)). However, we assume that the effect of mutation on the probability of replication is inverted in the recipient host (i.e. virions possessing all five mutations were assumed to have the highest fitness (figure 2*a*)). Thus, the probability of replication in the recipient host is the same as equation (2.2), but 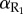 and 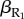 are replaced with 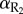 and 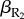 (electronic supplementary material, table S1).

We also explored two modes of host adaptation (figure 2*b*). First, we investigated ‘off-the-shelf’ adaptation in which all the mutations necessary for adaptation to a novel host must be transmitted from the donor host; hence, new mutations are unable to arise during replication in the recipient host. This is equivalent to saying that a virus population in the recipient is subject to selection but no new mutation. Second, we explored the ‘tailor-made’ model in which virions are able to accrue new mutations through replication in the recipient host (at mutation rate *m*).

### (f) Simulation experiments

The model was programmed in C++. We explored a range of *b* values (electronic supplementary material, table S1) with fitness functions *f*_1_ through *f*_8_, and assuming both off-the-shelf and tailor-made adaptation. We ran the model 200 times for each parameter combination. From each model run we recorded the diversity during the bottleneck, the dynamics of population growth in the recipient immediately following transmission, and the population size and distribution of genotypes after the specified period of infection in the recipient host.

## 3. Results

### (a) Probability of establishing infection in a new host

The probability of establishing infection in a new host is largely driven by the fitness effect, which dictates the probability that a specific virion type will reproduce. For the purposes of this study, we only evaluate if an infection becomes established in a new host and define an ‘established’ infection as one in which the virus population at the peak of infection (i.e. after 5 days of infection) in the recipient reaches > 1% of the carrying capacity (i.e. >1 000 000 virions).

**Figure 3.**
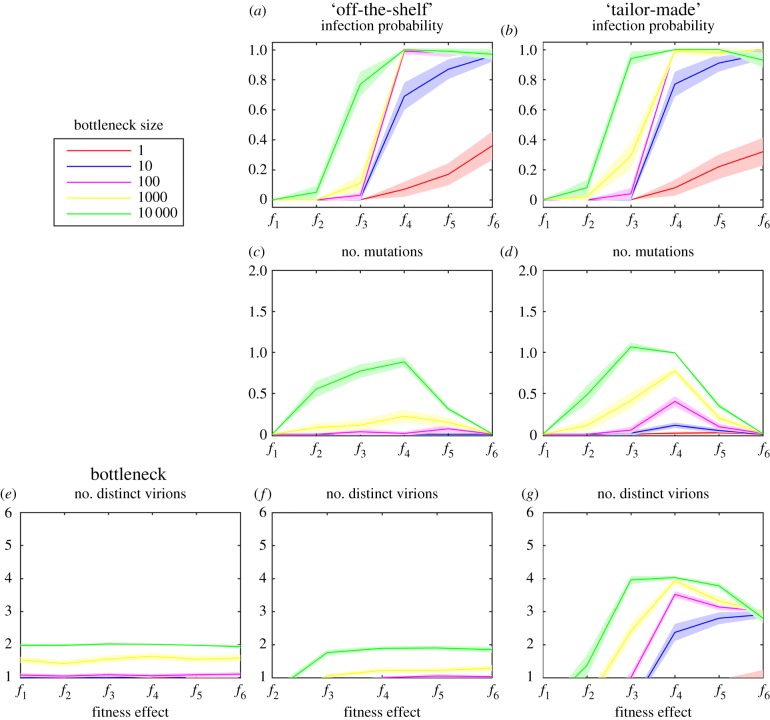
(*a*,*b*) The mean probability of establishing infection in a new host for each model—that is, the virus population in the recipient at the peak of infection (i.e. after 5 days of infection) is greater than 1% of the carrying capacity (i.e. population size is greater than 1 000 000 virions); (*c*,*d*) the mean number of mutations per virion for each model; and the number of distinct virus types in the transmission bottleneck (*e*) as well as in the virus population following infection in the recipient (*f*,*g*) for each model. All plots show the mean of established infections (solid line) and the 95% CI (shaded area) out of 200 simulations for increasing bottleneck sizes and different fitness effects. (Online version in colour.)

Both the off-the-shelf and tailor-made models showed very similar results under each parameter combination (figure 3*a,b*). For instance, under a strong fitness effect (i.e. *f*_1_), the fitness valley between two host species is very steep, such that there is a very low probability of establishing an infection. However, as the fitness valley becomes shallower, infection in the recipient host becomes more likely. In addition, the probability of infection is also increased with a more relaxed (i.e. looser) population bottleneck at transmission. An important difference between the two models was observed when the bottleneck *b* > 100 and when the fitness valley was moderately steep (i.e. *f*_3_): under this parameter space there was an increased probability of established infection under the tailor-made model. Overall, however, it is notable that there is a high probability of infection in the recipient host for a wide range of parameters, even if it is only transient (i.e. that the virus is not fully adapted to the new host).

We observed the population growth of each type of virion (i.e. virus genomes with *n* mutations, where *n* = 0–5) over the first 10 generations following transmission to the recipient host (electronic supplementary material, figure S1). Notably, under fitness function *f*_2_ the number of wild-type virions decreased while the number of mutated virions increased; hence, this might represent the point in parameter space that best facilitates adaptation and in turn the best chance of sustained emergence in a new host species.

### (b) Mean number of mutations per virion

The mean number of mutations per genome in the virus population was determined for different fitness effects and transmission bottlenecks. Under both models, the extent of positive selection (adaptive evolution) and bottleneck size had a considerable effect on the number of mutations per genome (figure 3*c,d*). Specifically, in the absence of strong positive selection (i.e. *f*_6_) or under a very steep fitness valley (i.e. *f*_1_), only wild-type variants were present in the population at all bottleneck sizes. Similarly, when the bottleneck was severe (*b* ≤ 100), the vast majority of genomes contained only wild-type variants. However, when selection was neither too weak nor too strong (*f*_2_–*f*_5_), a larger proportion of virions contained a higher number of mutations for a wide range of bottleneck sizes, such that this represented the optimal conditions for host adaptation. Under the tailor-made model, there were, on average, more mutations per virion for each parameter combination than under the off-the-shelf model.

### (c) Diversity of virus population in established infections

Owing to the large number of possible genotypes, we define genetic diversity simply as the number of distinct phenotypes in the population, assuming that virions possessing *n* mutations have equivalent fitness and thus phenotype. As expected, there are more distinct virions in the bottleneck population when the transmission bottleneck is wide (figure 3*e*). The maximum number of distinct virions under these relaxed parameters was, on average, approximately 2. Surprisingly, diversity in the bottleneck population was largely unaffected by the fitness effect. This lack of diversity is likely due to the very small proportion of the population being sampled, as well as selection acting against mutations in the donor.

We compared the number of distinct virions in the recipient host (figure 3*f,g*), which revealed major differences between the tailor-made and off-the-shelf models. In particular, more diversity was present after infection under the tailor-made model as the virus can adapt to its new host. Under this model, a greater number of distinct virion types were present when the fitness valley was not too steep and the bottleneck was loose. By contrast, the diversity under the off-the-shelf model largely resembled that present in the bottleneck population, such that very little adaptation has occurred as transmission, thereby highlighting the limits of this model of viral emergence.

### (d) The duration of viral replication

The duration of viral replication was varied from 6 h (i.e. 20 viral generations, over a 5-day infection) to both 8 and 12 h (i.e. 15 and 10 viral generations, respectively). Extending the duration of viral replication to 8 h had very little overall effect for either model (electronic supplementary material, figure S2*a*). By contrast, when viral replication took place over 12 h we found that there was (i) a decreased probability of infection for a wider range of parameters, (ii) fewer mutations and thus less adaptation to the recipient host and (iii) less overall diversity (electronic supplementary material, figure S2*b*). Very similar results were obtained for 12 h replication under both the off-the-shelf and tailor-made models.

### (e) Varying fitness effects between hosts

**Figure 4.**
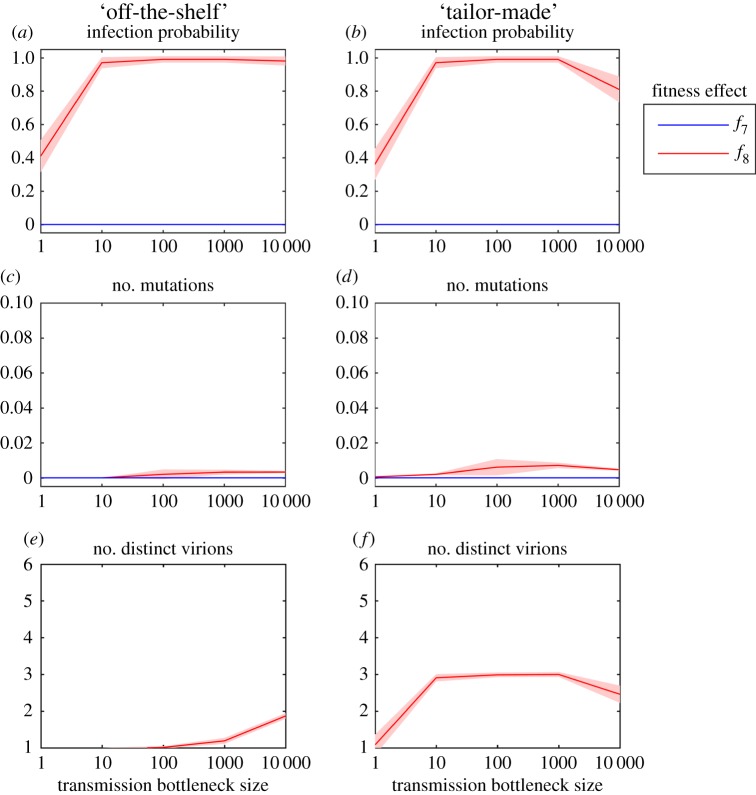
(*a,b*) The mean probability of establishing infection in a new host for each model—that is, where the virus population in the recipient at the peak of infection (i.e. after 5 days of infection) is greater than 1% of the carrying capacity (i.e. population size is greater than 1 000 000 virions); (*c,d*) the mean number of mutations per virion for each model and the number of distinct virus types in the virus population following infection in the recipient (*e,f*) for each model. All plots show the mean (solid line) and the 95% CI (shaded area) of established infections out of 200 simulations as a function of bottleneck size for two different fitness effects (*f*_7_ and *f*_8_). (Online version in colour.)

It is conceivable that viruses are inherently more likely to replicate in a new host (i.e. ‘generalists’), regardless of how constrained they might be in the original host. We, therefore, investigated two additional fitness functions, *f*_7_ and *f*_8_, that reflect differences in fitness effects between the hosts (figure 2*a*). Under *f*_7_, viral mutations in the donor host have very little effect on fitness (equivalent to *f*_6_), but a large effect once transmitted to the recipient host (equivalent to *f*_1_). By contrast, *f*_8_ represents the opposing case (i.e. *f*_1_ in the donor and *f*_6_ in the recipient). Under fitness scenario *f*_7_, we found that an infection is never established under either emergence model and for all parameter combinations (figure 4), likely due to the strong selection against wild-type virions in the recipient. Conversely, when the virus is a ‘generalist’ in the recipient (i.e. *f*_8_), there is an increased probability of infection, a higher number of mutations and a greater number of different virus types for both models (figure 4). In fact, under this scenario, there is a high probability that an infection will be established in the recipient host, even with a very severe transmission bottleneck.

## 4. Discussion

We investigated the likelihood of emergence under two models of host adaptation, nicknamed the ‘off-the-shelf’ and ‘tailor-made’ models. In both scenarios, the virus population in the donor host was subject to purifying selection such that any mutations that arose within this host had negative fitness effects. Nevertheless, error-prone virus replication may provide opportunities for multiple mutations to accumulate within a single viral genome even when they are deleterious. When transmitted from a donor species to a new recipient species, new genetic variants may result in a virus that is better adapted to the new host. Similar to previously proposed frameworks [7,30], virions not fully adapted to their host always have some adverse fitness effects. Under both models studied here, pathogen emergence is dependent on the strength of positive selection on advantageous variants that enable the virus to adapt to the new host over the course of an infection, thereby allowing onward transmission among the recipient population. We explored a wide range of fitness effects, which, in terms of the adaptive landscape, can be seen as a measure of the valley between two adaptive peaks. As the gradient of the valley increased, genetic variants were subjected to stronger opposing selection between the donor and the recipient hosts.

Overall, our analysis clearly shows that the interactions between within-host and between-host fitness landscapes play a central role in determining the probability of emergence, analogous to earlier findings [7]. Under our implementation of the off-the-shelf model the probability of transmitting existing genetic variants by chance is greatly facilitated by a ‘very loose’ transmission bottleneck, observed here to be one comprising more than 0.01% of the virus population. Once transmitted, these genetic variants may increase in frequency under strong positive selection. If the fitness valley is too shallow or too steep, genetic variants remain at frequencies that are too low to enable adaptation, even when transmitted through a very loose bottleneck. Therefore, under this model, both a loose bottleneck and moderately strong positive selection represented the optimal conditions for host adaptation. Conversely, under the tailor-made model, favourable mutations that are subject to positive selection can occur during the recipient's infection, such that selection is the main driver of emergence. Nevertheless, when the fitness valley is too steep (i.e. many mutations and/or a large fitness effect), emergence is greatly hampered. Additionally, in marked contrast with the off-the-shelf model, the virus may generate a higher number of mutations, as is required for adaptation in the recipient host even when the bottleneck is severe (i.e. approx. 100 virions).

The size of the transmission bottleneck has a dramatic effect on the population dynamics of the evolving virus [31–33]. Attempts to estimate the size of real-world transmission bottlenecks outside of plants have largely been limited to studying transmission between animal models within a laboratory setting. In human infections the size of transmission bottlenecks has generally been inferred from intra-host genetic diversity by examining known chains of transmission. For example, in the case of human influenza A virus, examining the transmission events within and across households revealed the transmission of multiple genetic variants between individuals, suggestive of a relatively loose bottleneck [34]. By contrast, for HIV infections, 60–90% of transmissions contain just a single genotype, in marked contrast with the high levels of diversity normally observed within hosts [35]. In addition, very limited genetic diversity was seen in mammals compared with birds infected with H7N9 avian influenza virus [36], compatible with the occurrence of a severe transmission bottleneck. However, our modelling implies that even with a moderate bottleneck size of approximately 100 virions, very low levels of genetic diversity may exist in the viral population during transmission from the donor to the recipient. This lack of genetic diversity is most likely because genetic variants in the donor are frequently deleterious, and therefore, purged by purifying selection such that they have not been sampled through the bottleneck. It is also possible that multiple beneficial mutations are competing with one another (i.e. clonal interference) which will also reduce genetic diversity [37]. Hence, an observed lack of genetic diversity in a virus population sampled following inter-host transmission does not necessarily equate with the occurrence of a severe population bottleneck. Even under a shallow fitness valley, we observed that genetic diversity remained low, presumably because the infection period was too short to enable sufficient genetic variation to accumulate and be sampled by chance.

In the case of influenza, data regarding viral aerosol shedding from infected individuals may be especially informative. Milton *et al.* [38] sampled exhaled particles from 37 patients within 5 days of onset of seasonal influenza and measured viral copy number using quantitative RT-PCR. Without the use of a facemask, the median number of viral copies of fine particles (less than or equal to 5 µm) exhaled was 560, with a geometric mean of 110 (95% CI: 45–260). While the median number of coarse viral particles (greater than 5 µm) was below the limit of detection, there were 37 viral copies in the 75th percentile, with a geometric mean of 12 (95% CI: 4–37). In addition, the study provided estimates of the infectiousness of the exhaled aerosols and detected viral RNA in 92% of the fine particle samples and 43% of the coarse particle samples. On this basis, and further assuming that only a proportion of exhaled virions are successfully transmitted to a new host (such that a bottleneck size of approx. 10–100 viral particles does reflect real-world parameters), we can conclude that infection may be established under the tailor-made model, but only when the fitness valley is neither too steep nor too shallow. Under the same assumptions it is similarly possible to conclude that emergence is unlikely to occur under the off-the-shelf model with any of the parameters investigated here.

As few as five mutations may be required for avian H5N1 influenza virus to evolve airborne transmission in mammals [2]. Using H5N1 as a model, we can define the probability of successful emergence as the proportion of virions that posses all five mutations required for onward transmission in the new host species. The results of our model suggest that the sustained emergence of an H5N1-like virus is extremely unlikely to occur under either model. Nevertheless, these results indicate where in parameter space host adaptation might be more likely. Indeed, analogous models of within-host adaptation found high viral loads containing one and two mutations following infection, although four or more mutations were unlikely to evolve [39]. The present model also reiterates findings from other theoretical studies that the within-host fitness landscape is dominant in shaping the probability of emergence [3], particularly in the initial host where potential evolutionary trajectories are likely to be determined [7]. The current model provides a framework that may be altered to incorporate additional parameter settings. For example, it is possible that our results are sensitive to a 5-day infection, particularly as it been suggested that an extended duration of infection that may occur within immunocompromised hosts, may be central to the generation and transmission of those mutations required for the emergence of a pandemic virus [4,39,40].

An additional concept to consider in this context is that the mutations required to enable mammalian transmission vary in their fitness effect. In particular, it is possible to conceive a two-step process in which a mutation of major effect enables a leap across a steep fitness valley, followed by the later accumulation of additional mutations of minor effect that marginally optimize or reduce viral fitness in the recipient and donor, respectively [5]. Under this scenario, a mutation of major effect may allow virions to cross the valley between two adaptive zones and thus achieve a higher overall fitness in a novel host. Herein, we have explored a wide range of fitness effects for two distinct models of host adaptation and the framework we propose can be easily adjusted and implemented for a variety of pathogens, accounting for different rates of mutation, replication and transmission.

It is unclear which model described here most accurately describes the process of pathogen emergence in nature. Indeed, it is important to note that little is known about where in parameter space the real-world settings of virus evolution and transmission lie. In addition, ecological factors too broad to parametrize here, such as increasing land-use, urbanization and climate change, will, unquestionably, have a considerable influence on the emergence of new diseases [25], particularly by changing the proximity of donor and recipient species. Nevertheless, by modelling biological dynamics of an evolving virus jumping between species we have revealed viral population-level barriers to host adaptation that are otherwise difficult to establish experimentally. Our results give an indication under which parameter settings the host adaptation of a novel virus, such as the emergence of H5N1 influenza virus in mammals, may be possible.

## Supplementary Material

Supplementary Table 1

## Supplementary Material

Supplementary Figure 1

## Supplementary Material

Supplementary Figure 2

## Supplementary Material

The Model

## Supplementary Material

READ ME.txt

## Supplementary Material

RUN.txt

## Supplementary Material

Source.txt

## Data accessibility

The datasets supporting this article have been uploaded as part of the electronic supplementary material.
