# Supplementary material for: Pathogen population bottlenecks and adaptive landscapes: overcoming the barriers to disease emergence
Source: Proc Biol Sci. 2016 Aug 31;283(1837):20160727. doi: 10.1098/rspb.2016.0727 (PMC5013787; doi:10.1098/rspb.2016.0727)
Supplement: Supplementary Table 1 [file rspb20160727supp1.docx]

**Supplementary Table 1.** Model parameters and variables, their notation, effect and value.

| **Parameter / Variable** | **Notation** | **Explanation** | **Value** |
| --- | --- | --- | --- |
| Mutation rate | *m* | The probability of one locus mutating from one state to the other (either wild-type, 0, or mutated, 1) | 0.001 |
| Number of Mutations | *n* | The number of mutated loci a virion carries | Variable (0 to 5) |
| Bottleneck Size | *β* | The number of founders in the recipient host | 1, 10, 100, 1000 or 10000 |
| Survival Intercept | α*_S_* | The intercept in a logistic regression giving the probability of survival as function of density. Equivalent to log odds of survival when density is 0. |  |
| Survival Slope | *β_S_* | The slope in a logistic regression giving the probability of survival as function of density; how log odds of survival changes with density |  |
| Population Density | *P* | The total number of virions divided by the carrying capacity (10^7^) | Variable (0 to 1) |
| Number of Virions at Replication | *r* | The number of virions that arise when a single virion replicates. | 16 |
| Replication Intercept, donor host | α*_R_*_1_ | The intercept in a logistic regression giving the probability of replicating as function of genotype in the donor host | 6 |
| Replication Slope, donor host | *β_R_*_1_ | The slope in a logistic regression giving the probability of replicating as function of genotype in the donor host | *f*_1_ = -2.5; *f*_2_ = -1.75; *f*_3_ = -1.5; *f*_4_ = -1.3125; *f*_5_ = -1.125; and *f*_6_ = -0.825. |
| Replication Intercept, recipient host | α*_R_*_2_ | The intercept in a logistic regression giving the probability of replicating as function of genotype in the recipient host | *f*_1_ = 1.875; *f*_2_ = 0.375; *f*_3_ = -0.5625; *f*_4_ = -1.5; *f*_5_ = -2.75; and *f*_6_ = -6.5. |
| Replication Slope, recipient host | *β_R_*_2_ | The slope in a logistic regression giving the probability of replicating as function of genotype in the recipient host | *f*_1_ = 2.5; *f*_2_ = 1.75; *f*_3_ = 1.5; *f*_4_ = 1.3125; *f*_5_ = 1.125; and *f*_6_ = 0.825. |
