# Supplementary figures and images for: Pathogen population bottlenecks and adaptive landscapes: overcoming the barriers to disease emergence
Source: Proc Biol Sci. 2016 Aug 31;283(1837):20160727. doi: 10.1098/rspb.2016.0727 (PMC5013787; doi:10.1098/rspb.2016.0727)

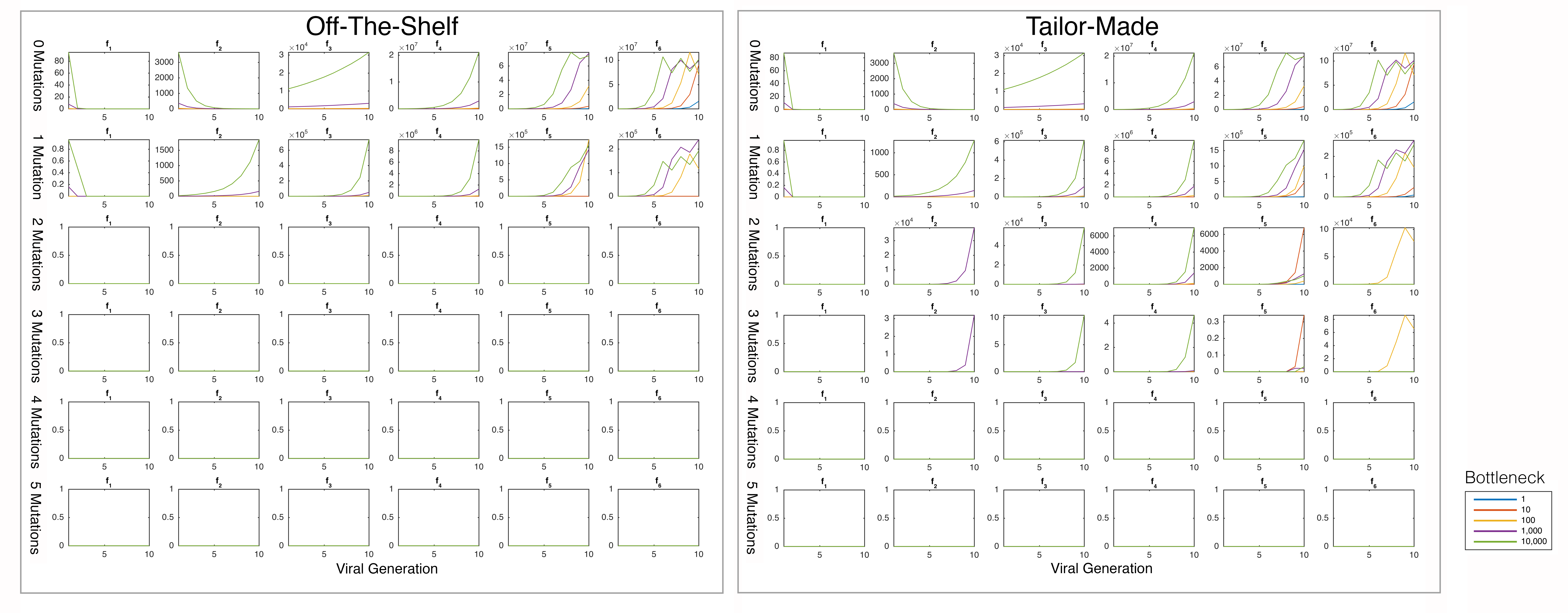

Supplement: Supplementary Figure 1 [file rspb20160727supp2.tif]

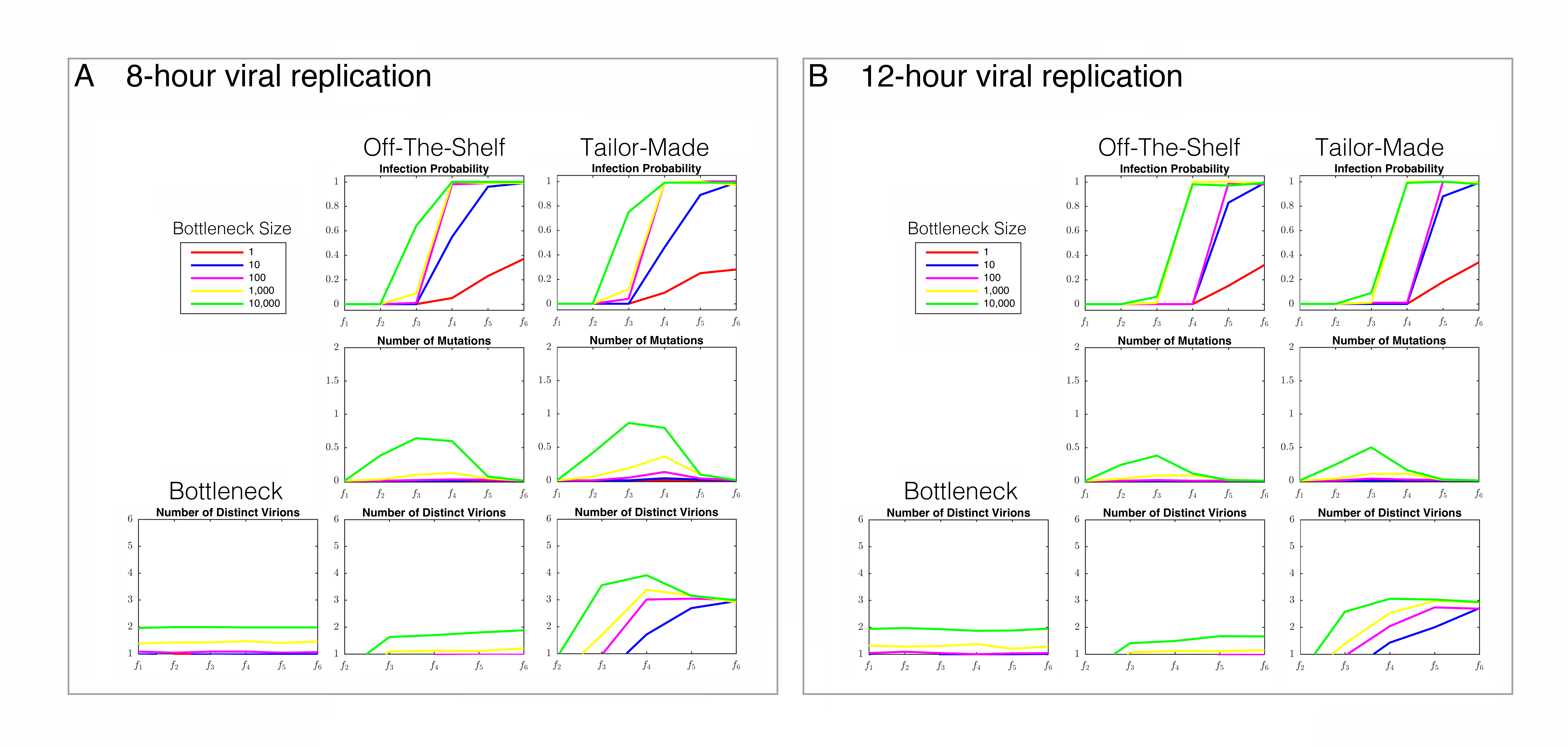

Supplement: Supplementary Figure 2 [file rspb20160727supp3.tif]
